# Risk factors for lower respiratory tract infection in children with tracheobronchial foreign body aspiration

**DOI:** 10.1097/MD.0000000000014655

**Published:** 2019-03-08

**Authors:** Bing Zhong, Si-Lu Sun, Jin-Tao Du, Di Deng, Feng Liu, Ya-Feng Liu, Liu Shi-Xi, Fei Chen

**Affiliations:** a Department of Otolaryngology Head and Neck Surgery, West China Hospital, Sichuan University; b State Key Laboratory of Oral Diseases and National Clinical Research Center for Oral Diseases and Department of Oral Medicine of West China Hospital of Stomatology, Sichuan University, Chengdu, Sichuan, China.

**Keywords:** antibiotic, children, hospitalization, lower respiratory tract infection, multivariate analysis, tracheobronchial foreign body aspiration, univariate analyses

## Abstract

The aim of this study was to determine the risk factors for lower respiratory tract infection (LRTI) in children caused by tracheobronchial foreign body aspiration (TFBA).

A total of 351 patients were retrospectively reviewed; all patients were diagnosed with TFBA at West China Hospital of Sichuan University from 2015 to 2017. Univariate analyses and multivariate analysis were used.

Age (<2 years) (*P* < .001), type of foreign body (plant) (*P* < .001), shape of foreign body (nonsmooth) (*P* < .001), and residence time of foreign body (>7 days) (*P* = .001) were risk factors for LRTI on univariate analysis. Multivariate analysis showed age (<2 years) (hazard ratio [HR] = 4.457; 95% confidence interval [CI] = 2.031–6.884; *P* < .001), type of foreign body (plant) (HR = 2.686; 95% CI = 1.577–3.452; *P* < .001), shape of foreign body (nonsmooth) (HR = 1.649; 95% CI = 1.437–3.663; *P* < .008), and residence time of foreign body (>7 days) (HR = 1.751; 95% CI = 1.329–3.554; *P* = .004) were independent risk factors for LRTI. Furthermore, children with LRTI also had longer lengths of hospital stays and antibiotic use than did children without LRTI.

Age, plant foreign body, nonsmooth foreign body, and long-term incarceration were all independent risk factors for LRTI in children. These results can help us to select more appropriate intervention times and stratified treatment for children with TFBA.

## 1 Introduction

Tracheobronchial foreign body aspiration (TFBA) is a common clinical emergency, with symptoms including hoarseness, cough, and dyspnea, more likely to appear in children and possibly associated with immature teeth and uncoordinated swallowing.^[1,2]^ The anatomical structure of the right main bronchi makes foreign bodies more likely to be incarcerated.^[3]^ Lateral bronchi are straighter and thicker than the left bronchus, facilitating the deposit of foreign bodies.^[4,5]^ Patients with mild symptoms may delay hospital visits for longer periods of time, leading to more severe complications.

Foreign bodies include plant, animal, mineral, and chemical compounds. Generally, free fatty acids of plants cause substantial irritation to the airway, leading to mucosal congestion, swelling and secretion, making the procedure more difficult.^[6,7]^ Many children are often diagnosed with bronchitis because of the neglect of chest X-ray examinations.^[8]^ As a result, lower respiratory tract infection (LRTI) and emphysema have been found when TFBA are found. Pneumonia and bronchitis were the most common LRTI associated with TFBA.^[9,10]^ Although most patients are discharged the day after surgery, LRTI nevertheless poses a difficulty for clinical treatment. The purpose of our study was to evaluate the risk factors for LRTI in children with TFBA, providing a basis for clinical treatment.

## 2 Materials and methods

### 2.1 Patients

This study was approved by the ethics committee of West China Hospital. We retrospectively analyzed a total of 351 children diagnosed with TFBA in West China Hospital of Sichuan University from 2015 to 2017. Patients meeting the following inclusion criteria were selected: children under the age of 14; and foreign bodies identified and removed by rigid bronchoscopy under general anesthesia. Exclusion criteria were as follows: over 14 years old; respiratory infection or systemic infection before incarceration of foreign bodies; and children who died before treatment. At the time of admission, the duration of foreign body incarceration was determined by chief complaint, and the symptoms and signs of children were observed to determine the trident sign. Chest X-ray and computed tomography (CT) were used to determine whether there was emphysema. All bronchoscopy procedures to remove of the airway foreign body were performed by a single operator.

### 2.2 Definitions

LRTI is defined as infection and inflammatory reaction of the trachea, main bronchi, and the various bronchi of the lungs. LRTI radiological criteria include a clear chest radiograph (pneumonia) or bronchoscopy (bronchitis). Clinical findings must satisfy at least one of the following conditions: new and/or progressive and persistent respiratory symptoms, including cough and sputum; fever (axillary temperature >37.5°C) or body temperature is too low (axillary temperature <36.0°C); physical examination revealing reduced respiratory rate or wet rales; white blood cell count >10 × 10^9^/L or <4 × 10^9^/L; and a positive sputum culture. Three depressions signs were defined as follows: suprasternal fossa, superior clavicular fossa, and intercostal space are substantially depressed when inhaling. The foreign bodies are classified as plant, animal, mineral, and chemical compounds according to their properties. Smooth foreign bodies were defined as objects without distinct edges or angles. The remainders were referred to as nonsmooth foreign bodies.

### 2.3 Statistical analysis

A chi-squared test was performed to analyze the association between LRTI and each factor. Factors significant in the univariate analysis were used as covariates in the multivariate analysis that was performed using a logistic regression model. We used the Wilcoxon–Mann–Whitney test to analyze the association between LRTI and postoperative duration of hospitalization. All statistical analyses were performed using the Statistical Product and Service Solutions (SPSS) software version 22.0 (IBM Corporation, Armonk, NY). *P* < .05 was considered statistically significant.

## 3 Results

### 3.1 Baseline characteristics and clinical features of LRTI

The median age of our study was 1.2 years, including 171 males and 180 females (Table 1). There were 14 foreign bodies located in trachea, 92 in the left main bronchus, and 255 in the right main bronchus. There were 311 plant, 19 animal, 12 mineral, and 9 chemical foreign bodies. There were 98 smooth and 253 nonsmooth foreign bodies with 15 days median residence time. We found that in 96 patients there were 3 depressions sign and in 287 patients there was emphysema.

**Table 1:**
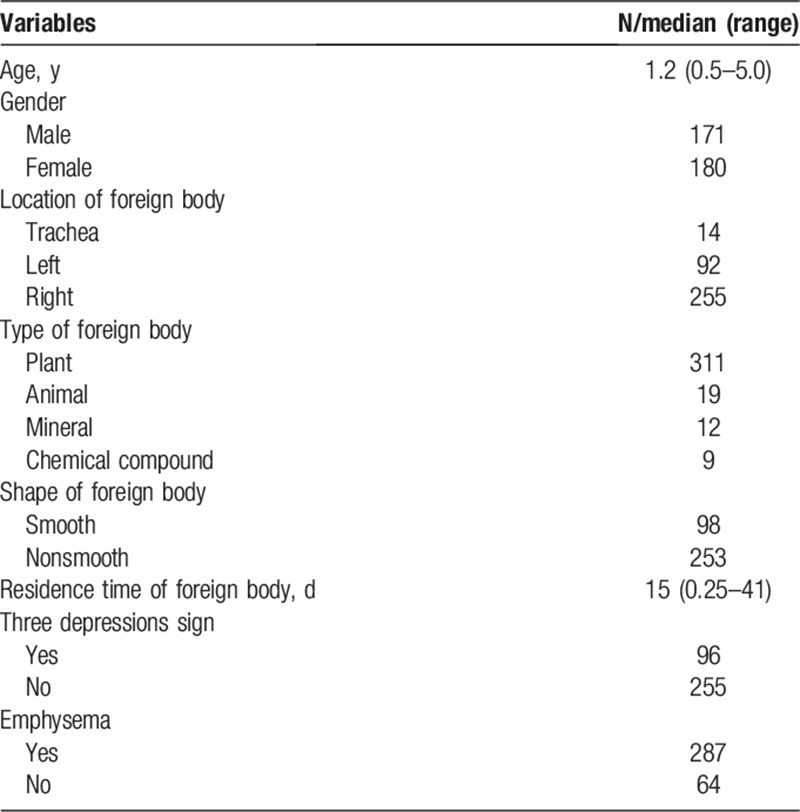
Patient characteristics.

### 3.2 Risk factors are associated with LRTI

As shown in Table 2, univariate analyses suggested that age (<2 years) (*P* < .001), type of foreign body (plant) (*P* < .001), shape of foreign body (nonsmooth) (*P* < .001), and residence time of foreign body (>7 days) (*P* = .001) were all potential contributors to LRTI. Multivariate analysis also indicated that age (<2 years) (hazard ratio [HR] = 4.457; 95% confidence interval [CI] = 2.031–6.884; *P* < .001), type of foreign body (plant) (HR = 2.686; 95% CI = 1.577–3.452; *P* < .001), shape of foreign body (nonsmooth) (HR = 1.649; 95% CI = 1.437–3.663; *P* < .008), and residence time of foreign body (>7 days) (HR = 1.751; 95% CI = 1.329–3.554; *P* = .004) increased the incidence of LRTI (Table 3).

**Table 2:**
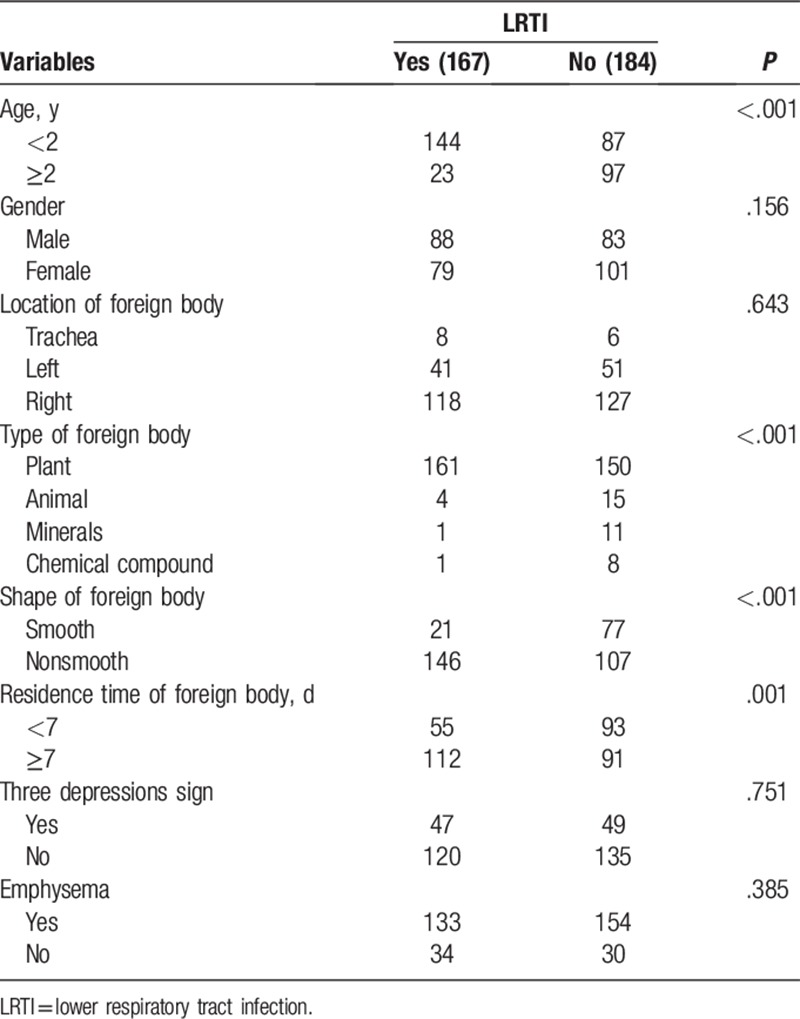
Univariate analysis of association between each factor and LRTI.

**Table 3:**
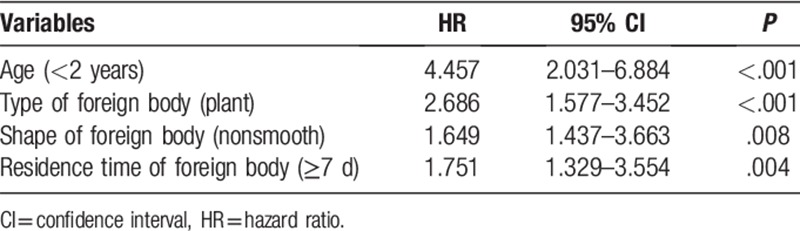
Multivariate analysis of factors associated with lower respiratory tract infection.

### 3.3 Association between LRTI and postoperative outcomes

**Figure 1:**
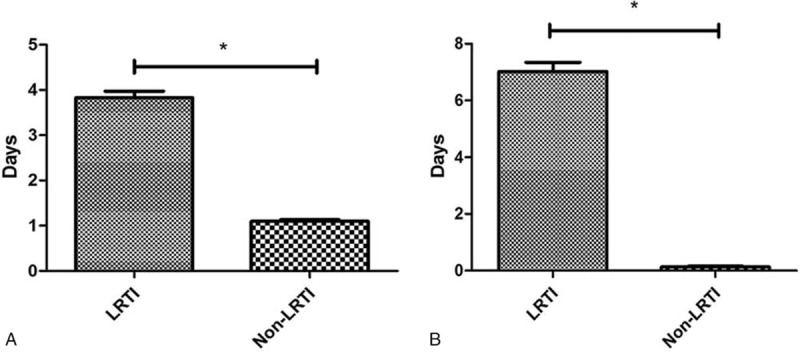
Association between LRTI and postoperative hospitalization. (A) The postoperative hospitalization time of children with LRTI was significantly longer than that of non-LRTI patients. (B) Postoperative antibiotic use time of children with LRTI was significantly longer than that of non-LRTI patients. LRTI = lower respiratory tract infection. ^∗^*P *< .001.

Postoperative hospitalization time of children with LRTI was significantly longer with that of non-LRTI patients (*P* < .001) (Fig. 1A), possibly related to complications from LRTI and longer duration of anti-inflammatory treatment. Furthermore, LRTI patients had significantly longer antibiotic use than non-LRTI patients (Fig. 1B).

## 4 Discussion

TFBA is an acute disease in children that may endanger lives; it often occurs in children aged 0 to 3 years, accounting for 65% to 75% of all cases.^[11,12]^ Early diagnosis and surgery are the keys to reducing mortality.^[4,13]^ Peanuts and sunflower seeds had been the most-reported foreign bodies in previous studies.^[14,15]^ Many children have already experienced complications such as emphysema and obstructive pneumonia when present to the hospital. Pathological reactions to foreign bodies vary according to the type of foreign body.^[16,17]^ Plants were related to local inflammatory reactions and sharp foreign bodies could easily lead to bleeding, emphysema, and pneumothorax.^[18,19]^ Sunflower seeds have been reported as a common tracheal foreign bodies, and most patients have a history of suffocation.^[20]^ Many studies have found that complications of airway foreign body were related to the type of foreign body and the duration of obstruction of foreign body. We found that age, plant foreign body, nonsmooth foreign body, and long-term incarceration were significantly related to LRTI, a factor that is crucial for preoperative evaluation of the inflammatory state and severity of disease.

In general, the anatomical features of the right bronchi increase the chance of foreign body incarceration.^[2]^ In fact, the right bronchus is more vertical and wider than the left bronchus, and more foreign bodies are found in the right bronchus.^[21]^ In our study, incarceration was more common in the right main bronchus than other parts of the respiratory tract, as shown in previous studies. Although tracheal foreign bodies are rare, timely treatment is necessary because of the high mortality rate. Symptoms of tracheal foreign bodies are more severe than those of bronchial foreign bodies, and tracheotomy may be performed if necessary to save lives.^[22]^

The pathological process of TFBA is divided into the entry stage, the asymptomatic stage, the symptom recurrence stage, and the complication stage. LRTI primarily occurs during the complication period.^[11,23]^ Cough, dyspnea, and fever are common symptoms of TFBA. The severity of symptoms is related to the type of foreign body, incarceration position, and duration.^[4,14]^ Recent studies have shown that surface smoothness, oil release state, and retention time are associated with local granuloma formation.^[24]^ A study of 223 children with TFBA found that symptoms and foreign body types were associated with respiratory complications.^[11]^ Some children have symptoms that are not obvious and may be misdiagnosed as chronic bronchitis, thus delaying proper treatment for long periods. The key to the diagnosis of this disease lies in the history of foreign body inhalation.^[25,26]^ Preoperative assessment of a child's physical condition and severity of illness is critical for surgery.^[20]^ Furthermore, preventing children from crying can reduce the chance of foreign bodies moving, causing sudden death from asphyxiation. Complications such as LRTI, emphysema, heart failure, and pneumothorax make surgery more difficult.^[27]^ Antibiotics are necessary when infections occur; however, long-term use of antibiotics can lead to antibiotic resistance. It is very important for children to reduce the use time and dose of antibiotics while ensuring their efficacy. In our study, we found that LRTI influenced hospitalization time. This may be because postoperative complications require longer antibiotic treatment. TFBA should be classified more clearly based on the risk factors of the LRTI to provide different management programs and treatments to improve prognosis and reduced hospitalization times.

Chest X-ray and CT are routine examinations, both of which have strong specificity for TFBA. It is important to note, however, that chest X-ray may be normal within 24 hours and that for children who are unable to describe their condition effectively, chest X-ray alone may not make a definitive diagnosis.^[28]^ Previous studies have shown that CT has higher specificity and sensitivity than chest X-ray. Chest CT could more clearly determine whether a foreign body obstructs the airway, and may identify related complications such as emphysema and atelectasis.^[29]^ However, the physical properties of inhaled materials limit CT's ability to detect disease. Chest CT scans more easily detect metal and bone foreign bodies than plants or plastic.^[30]^ Bronchoscopy is necessary for patients with unclear diagnoses. Rigid bronchoscopy and electronic bronchoscopy are both suitable for localization of foreign bodies in the trachea and deep bronchi.

TFBA is related to physiological and psychological development and to family care.^[2]^ Children's curiosity often leads them to habitually put strange things their mouths, increasing the risk of TFBA.^[31]^ The poor chewing function of children is also an important cause of TFBA. The incomplete development of molars in children aged 2 to 3 often leads to insufficient chewing of food, also greatly increasing the incidence of TFBA.^[32]^ Rural children contribute more than 95% of TFBA in China.^[33]^ The numbers of left-behind children are increasing due to the outflow of the labor force in China, as well as the increased proportion of grandparents as guardians of children. The low education level of grandparents leads to irregular diets and increases the chances of exposure to small foreign bodies in children. Particularly in the countryside, the lack of popularization of disease mechanisms and first-aid methods leads to a large number of complications and death from TFBA.

Interestingly, many studies have found that male children experience more TFBA than do female children with a ratio of 2:1.^[4,28]^ The carelessness and naughtiness of boys leads to a higher risk of TFBA.^[11]^ A 20-year retrospective study of TFBA in children suggests that the number of cases in boys was much higher than in girls, 2079 versus 1070, respectively.^[33]^ Sumanth et al reported that males were more commonly affected than were females (2.7:1).^[14]^ However, Baram et al reported that among the 83 TFBA children, the number of female and male children were 43 (51.8%) and 40 (48.2%), respectively.^[20]^ In our study, there was a similar ratio of boys to girls, inconsistent with some of these previously mentioned reports. The backwardness of western China results in the low degree of education for most guardians which make the supervision deficits and low levels of understanding regarding related TFBA in guardians. Naughty boys may account for a small proportion of the causes of TFBA, which results in the similar proportion of boys and girls with TFBA. In addition, a small sample size may also contribute to this result.

This study has some limitations. This was a single-center retrospective study, limited to LRTI for TFBA patients. For the large number of TFBA patients in China, the number of patients we studied was small. In addition, we did not provide more detailed age groups for children of different ages. Among our patients, ethnic differences may also cause bias (Tibetan and Han). Education background plays an important role in TFBA, this limitation in our analysis may lead to inaccurate results. Although the surgical method was the same, the use of different types of antibiotics has an impact on the results. Therefore, larger sample size and more detailed grouping need to be supplemented in the future.

## 5 Conclusion

We found that age, plant foreign body, nonsmooth foreign body, and long-term erosion were closely related to LRTI of TFBA. Paying attention to these risk factors may help to assess disease status and improve the prognosis of TFBA in children.

## Author contributions

Liu Shi-Xi and Fei Chen proposed the study. Bing Zhong and Si-Lu Sun performed the research and wrote the first draft. Bing Zhong and Si-Lu Sun collected and analyzed the study data. All authors contributed to the design and interpretation of the study and to further drafts. The first author of this manuscript is Bing Zhong and Si-Lu Sun.

**Conceptualization:** Si-Lu Sun.

**Data curation:** Si-Lu Sun, Jin-Tao Du, Di Deng, Feng Liu.

**Formal analysis:** Bing Zhong, Di Deng, Feng Liu, Ya-Feng Liu, Liu Shi-Xi, Fei Chen.

**Investigation:** Bing Zhong.

**Software:** Bing Zhong, Jin-Tao Du, Fei Chen.

**Writing – original draft:** Bing Zhong.

**Writing – review & editing:** Ya-Feng Liu, Liu Shi-Xi, Fei Chen.
